# Blood biochemistry of olive ridley (*Lepidochelys olivacea*) sea turtles foraging in northern Sinaloa, Mexico

**DOI:** 10.1371/journal.pone.0199825

**Published:** 2018-07-25

**Authors:** B. A. Espinoza-Romo, J. C. Sainz-Hernández, C. P. Ley-Quiñónez, C. E. Hart, R. Leal-Moreno, A. A. Aguirre, A. A. Zavala-Norzagaray

**Affiliations:** 1 Instituto Politécnico Nacional, CIIDIR- SINALOA, Guasave, Sinaloa, México; 2 Grupo Tortuguero de las Californias A.C., La Paz, B.C.S., México; 3 Investigación, Capacitación y Soluciones Ambientas y Sociales A.C. Tepic, Nayarit, México; 4 Department of Environmental Science and Policy, George Mason University, Fairfax, Virginia, United States of America; University of Minnesota, UNITED STATES

## Abstract

Blood parameters provide an excellent tool to evaluate the health status of wildlife. However, there are few studies about health parameters of sea turtles in Mexico. For olive ridley turtles (*Lepidochelys olivacea*), no information was available to establish the health baseline for the species. The objective of this study was to establish reference blood biochemistry values for olive ridley turtles in the northern Sinaloa foraging area. Between 2013 and 2015, 82 olive ridley turtles were captured. Body condition index (BCI) presented a mean of 1.46 ± 0.14 (1.17–2.02) that categorized the population with excellent body condition; in addition, 99% of the turtles captured had a good physical appearance. Blood was collected for biochemistry analysis from 60 turtles. Significantly higher values of total protein, albumin, A/G ratio (albumin/globulin) and PCV (packed cell volume or hematocrit) were observed in adult when compared to subadult turtles. On the other hand, no significant differences were found when females and males were compared. Based on the BCI, physical assessment, and blood parameters, and compared to other sea turtle species, olive ridley turtles in northern Sinaloa were considered in excellent health. To the best of our knowledge, this is the first study to establish normal blood biochemistry values of foraging olive ridley turtles in northern Sinaloa.

## Introduction

Clinical parameter studies are an essential tool in evaluating sea turtle health status as they allow the establishment of relationships regarding changes within these organisms and their environment [1]. Blood characteristics reflect not only physiological conditions but also ecological distinctions and characteristics for each species; these allow for the detection of alterations in an animal’s metabolic activity. These biomedical analyses are an essential tool that when combined with clinical history and physical examination, provides a picture of an individual’s health status [2]. However, this is only possible once species baseline biochemistry values have been established [3]. This knowledge is vital in helping to improve species management and conservation plans [4–6].

In addition, the establishment of reference values in sea turtles, should be carried out regionally [4], and reflect criteria such as age [7, 8], sex [9], diet [10] and reproductive status [11, 12]. Furthermore, only healthy individuals should be considered in the analysis [3].

Olive ridley turtles (*Lepidochelys olivacea*) are the most abundant sea turtle species worldwide. However, there is a lack knowledge regarding their health status. Due to pelagic habits, dead and stranded turtles have supplied much of the known information on their health parameters. Studies that do exist on live turtles have concentrated on individuals at nesting rookeries due to ease of access [13]. However, this only allows for sampling of nesting female turtles, whereas in foraging areas the population structure is better represented by animals of different sexes and life stages.

The objective of this study was to establish baseline blood biochemistry values for wild Sinaloa, Mexico olive ridley turtles. This information will contribute to the protection of the species and allow for long-term health assessment and monitoring.

## Materials and methods

### Study area

Turtles were captured in the marine area located 25 km west the San Ignacio-Navachiste-Macapule lagoon complex, Sinaloa (24° 50' and 25° 35' N and 108° 10' and 109 10' W). This area is recognized as an important sea turtle foraging ground due to the presence of high primary productivity [14]. Previous studies in this area have confirmed the presence of five of the seven sea turtle species known worldwide. Of these five species, the olive ridley is the most abundant [15, 16].

### Blood sampling

We collected blood samples between 2013 and 2015 during April-July, the months of highest olive ridley sea turtle abundance. Turtles were captured while they floated at the surface during thermoregulation. Simple random sampling was used and two standard capture techniques were employed: first, turtles were captured from the boat’s bow using a 1.5 meter metallic ring with nylon netting [17] and second, via the rodeo technique [18, 19].

Biometric data was taken according to the methodology described by Bolten (2000). Morphometric measurements were recorded including straight carapace length (SCL) using forestry callipers with 0.05 cm accuracy, and weight with a hanging spring scale with 0.1 kg accuracy [20]. Individual sea turtle age class was determined based on SCL, considering adults at ≥60 cm and subadult <60 cm [21]. Sex was determined in adults using tail length, *i*.*e*., long and thick tails in males and short tails in females; those with an absence of evident sexual dimorphism were classified as unknown [22].

Each turtle had a detailed physical examination including presence/absence of heavy epibiont loads, skin lesions including tumors, flipper amputations, miscellaneous abnormalities, emaciation and weakness [11, 23]. Body condition was assessed using the Fulton Body Condition Index BCI = $(\frac{weight}{{SCL}^{3}})*10000$ [24].

Blood samples were collected from the dorsal cervical sinus, using 21 gauge needles and 10 ml syringes and transferred into Vacutainer® tubes containing lithium heparin as an anticoagulant [25, 26]. Samples were stored under refrigeration at 4°C until laboratory processing. Each turtle was tagged on its posterior right flipper with a Monel tag (*National Band and Tag Company*) to avoid collection of data from a recaptured individual during sampling. After processing, all turtles were released in good condition.

### Laboratory analysis

Packed cell volume (PCV) was quantified using the microhematocrit method. A whole blood sample was placed in capillary tubes with lithium heparin as an anticoagulant agent and centrifuged for 5 min in a microcentrifuge model ZO-1 (*LW Scientific Inc*). We used plasma in preference to serum because in reptiles clot formation is unpredictable, changing biochemical values and occasionally producing haemolysis in the blood samples [27]. Plasma was separated using centrifuge COMPACT II model 420225 (*Becton Dickinson*) at 3000 rpm for 15 min and pipetted into 1.6-ml cryogenic vials. Plasma total protein was recorded using a small hand-held refractometer model VET-360 (*REICHERT*). We used 18 plasma parameters previously shown as the most representative for the establishment of sea turtle health profiles [28, 29]. Plasma samples were analysed using a chemistry analyser model VetTest 8008 (dry chemistry system) and commercial kits (IDEXX). Quality Control F1620 (IDEXX) was run periodically to verify that equipment was functioning correctly [30].

Analysed parameters were divided into three groups: 1) nutrients and metabolites–total protein (g dL^-1^), albumin (g dL^-1^), globulin (g dL^-1^), Albumin/Globulin (A/G) ratio, creatinine (mg dL^-1^), total bilirubin (mg dL^-1^), blood urea nitrogen (BUN) (mg dL^-1^), glucose (mg dL^-1^), cholesterol (mg dL^-1^) and triglycerides (mg dL^-1^); 2) enzymes—Alanine aminotransferase (ALT) (U L^-1^), aspartate aminotransferase (AST) (U L^-1^), alkaline phosphatase (ALKP) (U L^-1^), creatine phosphokinase (CK) (U L^-1^), gamma-glutamyl transpeptidase (GGT) (U L^-1^) and amylase (U L^-1^), and 3) electrolytes: calcium (mg dL^-1^) and phosphorus (mg dL^-1^). We excluded from our analysis blood samples that were insufficient (Plasma <210μL) or presented haemolysis.

### Ethics statement

Research permits were granted in México by Dirección General de Vida Silvestre / Secretaría de Medio Ambiente y Recursos Naturales -SEMARNAT (Mexican Wildlife Department of the Secretary of Environment and Natural Resources) as field permits SGPA/DGVS/00706/13, SGPA/DGVS/02259/14, and SGPA/DGVS/04478/15. These cover the sampling, collection and processing of blood samples. All sea turtles were released at the location of capture.

### Statistical analysis

Results are presented as mean, range and standard deviation (SD). Data normality and homoscedasticity (same variance) were assessed using a *Kolmogorov–Smirnov* and *Levene* tests, respectively. Differences in blood parameters between groups (age, sex and year) were assessed employing a one-way analysis of variance (*ANOVA*) and Tukey's multiple comparison test. The correlations among statistical variables were determined using a simple regression model (*R*^*2*^ *> 50%*) with *α = 0*.*05*. We performed all analyses using the statistical package Minitab® 17.1.0.

## Results

During 2013–2015, we captured 82 olive ridley turtles. We successfully obtained biochemical profiles from 60 of those turtles. We were unable to obtain blood samples from five turtles, and we discarded 17 samples due to haemolysis. No recaptures occurred during monitoring. Descriptive statistics of body condition (BCI), SCL, weight and blood parameters of olive ridley sea turtles are presented in Table 1.

**Table 1:** Morphometric and blood biochemistry values of olive ridley turtles (*Lepidochelys olivacea*) from a foraging aggregation in northern Sinaloa, Mexico, 2013–2015.

Parameter	Mean±SD	Range
Weight (kg)	31.2±5.0	17.0–44.0
SCL (Cm)	59.8±3.1	50.5–65.8
BCI	1.47±0.1	1.1–2.0
PCV (%)	30.3±7.6	19.0–52.0
Total protein (gdL^-1^)	3.9±0.6	2.7–5.9
Albumin (gdL^-1^)	1.0±0.2	0.6–2.0
Globulin (gdL^-1^)	2.9±0.4	1.6–3.9
A/G ratio	0.3±0.1	0.2–1.1
Total bilirubin (mgdL^-1^)	0.7±0.8	0.1–3.0
Creatinine (mgdL^-1^)	0.3±0.4	0.0–2.2
BUN (mgdL^-1^)	63.3±16.9	19.0–127.0
Glucose (mgdL^-1^)	122.6±34.8	62.0–224.0
Cholesterol (mgdL^-1^)	100.0±40.8	33.0–256.0
Triglycerides (mgdL^-1^)	195.2±100.2	43.0–375.0
ALKP (UL^-1^)	34.8±15.5	12.0–94.0
ALT (UL^-1^)	26.2±11.5	12.0–73.0
AST (UL^-1^)	143.6±118.6	28.0–392.0
GGT (UL^-1^)	0.01±0.12	0.0–1.0
CK (UL^-1^)	245.3±386.0	0–1537.0
AMYL (UL^-1^)	303.2±95.4	89.0–525.0
Calcium (mgdL^-1^)	4.6±2.8	0.0–10.7
Phosphorus (mgdL^-1^)	9.7±2.4	6.5–16.1

N = 60, SCL = Straight carapace length, PCV = Packed cell volume, A/G = Albumin/Globulin, BUN = Blood urea nitrogen, AMYL = Amylase, ALKP = Alkaline phosphatase, ALT = Alanine aminotransferase, AST = Aspartate aminotransferase, GGT = Gamma-glutamyl transpeptidase, CK = Creatine phosphokinase. Standard deviation (SD).

Most turtles were apparently healthy with 99% of animals presenting low or no epibiont load and absence of injuries and deformities [31]. The overall BCI value in captured olive ridley sea turtles was 1.46 ± 0.14. Individually, 99% of turtles were in excellent condition and 1% in very good condition [32].

With regard to life stage, adult turtles presented significantly higher concentrations of total protein (*p = 0*.*046*), PCV (*p <0*.*001*), albumin (*p <0*.*001*) and A/G ratio (*p = 0*.*05*) when compared to subadults (Table 2). We found no significant differences between blood parameters in males and females (*p>0*.*05*). Turtles sampled in 2013 presented significantly higher concentrations of total protein (*p = 0*.*014*), globulin (*p = 0*.*010*) and triglycerides (*p <0*.*001*) compared to turtles sampled in 2015. In 2015, glucose levels (*p <0*.*001*) and CK (*p <0*.*001*), AST (*p <0*.*001*) and AMYL (*p = 0*.*002*) enzymatic activity were significantly higher than in 2013 and 2014. Finally, PVC was significantly lower (*p = 0*.*013*) in 2015 when compared to previous years (Table 3). There were no correlations between BCI and analysed blood parameters.

**Table 2:** Morphometric and blood biochemistry values of adult and subadult olive ridley turtles (*Lepidochelys olivacea*) from a foraging aggregation in northern Sinaloa, Mexico, 2013–2015.

Parameter	Adults (*n* = 31)		Subadults (*n* = 29)	
	Mean±SD	Range (n^a^)	Mean±SD	Range(n^a^)
Weight (kg)	34.9±3.7	29.0–42.0	28.4±4.5	17.0–37.0
SCL (cm)	61.7±1.5	60.0–65.8	57.0±2.9	50.5–59.8
PCV (%)*	33.6±7.7ª	19.0–52.0 (26)	26.1±5.0^b^	20.0–35.0 (22)
Total protein (gdL^-1^)*	4.1±0.6ª	2.8–5.9	3.8±0.6^b^	2.7–5.2
Albumin (gdL^-1^)*	1.1±0.2ª	0.7–2.0	0.9±0.2^b^	0.6–1.5
Globulin (gdL^-1^)	2.9±0.5	1.6–3.9	2.8±0.4	2.1–3.7
A/G ratio*	0.4±0.1^a^	0.2–1.1	0.3±0.0^b^	0.2–0.4
Total bilirubin (mgdL^-1^)	0.8±0.9	0.1–3.0	0.4±0.1	0.2–0.6
Creatinine (mgdL^-1^)	0.3±0.4	0.0–2.2 (30)	0.3±0.4	0.1–1.9
BUN (mgdL^-1^)	66.9±20.3	44.0–127.0	59.5±11.3	19.0–78.0
Glucose (mgdL^-1^)	123.2±32.5	62.0–202.0	122.0±37.6	67.0–224.0
Cholesterol (mgdL^-1^)	107.6±44.6	39.0–256.0	92.2±35.5	33.0–171.0
Triglycerides (mgdL^-1^)	219.1±106.9	43.0–375.0	169.6±87.2	63.0–373.0
ALKP (UL^-1^)	32.0±12.6	12.0–64.00	37.8±31.1	15.0–94.0
ALT (UL^-1^)	29.0±12.9	12.0–73.0 (24)	23.0±8.8	12.0–40.0 (21)
AST (UL^-1^)	124.8±111.2	30.0–392.0 (29)	163.1±125.0	28.0–402.0
GGT (UL^-1^)	0.03±0.17	0.0–1.0	0.0±0.0	0.0–0.0
CK (UL^-1^)	236.5±403.4	0–1537.0	254.7±374.4	0–1205.0
AMYL (UL^-1^)	323.7±94.8	160.0–525.0 (28)	281.3±92.8	89.0–473.0
Calcium (mgdL^-1^)	4.2±2.8	0–10.7	5.1±2.7	0.1–9.8
Phosphorus (mgdL^-1^)	10.4±2.6	6.6–16.1 (25)	9.2±2.0	6.5–14.7

SCL = Straight carapace length, PCV = Packed cell volume, A/G = Albumin/Globulin, BUN = Blood urea nitrogen, AMYL = Amylase, ALKP = Alkaline phosphatase, ALT = Alanine aminotransferase, AST = Aspartate aminotransferase, GGT = Gamma-glutamyl transpeptidase, CK = Creatine phosphokinase. SD = Standard deviation

* = Significant difference

n^a^ = Number of samples analyzed if < n

differing letters indicate significant difference between groups.

**Table 3:** Morphometric and blood biochemistry values of olive ridley turtles (*Lepidochelys olivacea*) from a foraging aggregation in northern Sinaloa, Mexico by year of capture, 2013–2015.

Parameter	2013 (*n* = 17)		2014 (*n* = 23)		2015 (*n* = 20)	
	Mean±SD	Range (n^a^)	Mean±SD	Range (n^a^)	Mean±SD	Range (n^a^)
Weight (kg)	31.1±4.3	22.0–38.0	33.0±5.8	19.0–42.0	31.9±4.8	17.0–44.0
SCL (cm)	59.2±3.1	51.7–64.0	59.4±3.3	51.1–64.0	60.5±2.9	50.5–65.8
PCV (%)*	34.5±3.8^a^	29.0–40.0	32.7±8.7^ab^	20.0–52.0	26.7±5.6^b^	19.0–37.0
Total protein (gdL^-1^)*	4.3±0.3^a^	3.9–5.2	3.9±0.7^ab^	2.8–5.9	3.7±0.5^b^	2.7–5.2
Albumin (gdL^-1^)	1.1±0.1	0.9–1.6	1.1±0.3	0.6–2.0	0.9±0.2	0.6–1.5
Globulin (gdL^-1^)*	3.2±0.2^a^	2.8–3.7	2.8±0.5^b^	1.6–3.9	2.7±0.4^b^	2.1–3.7
A/G ratio	0.2±0.0	0.2–0.4	0.4±0.2	0.2–1.1	0.3±0.0	0.2–0.5
Total bilirubin (mgdL^-1^)	0.3±0.1	0.2–0.5	1.0±0.9	0.2–3.0	0.3±0.2	0.1–0.6
Creatinine (mgdL^-1^)	0.2±0.5	0–2.2.0	0.4±0.5	0.1–1.9	0.3±0.1	0.1–0.6 (19)
BUN (mgdL^-1^)	54.3±7.5	44.0–70.0	72.5±21.0	48.0–127.0	60.4±12.0	19.0–76.0
Glucose (mgdL^-1^)*	95.0±19.8^b^	62.0–125.0	124.9±30.0^a^	70.0–224.0 (22)	143.5±35.2^a^	68.0–202.0
Cholesterol (mgdL^-1^)	118.0±38.7	44.0–193.0	95.1±43.6	39.0–256.0	90.2±36.0	33.0–162.0
Triglycerides (mgdL^-1^)*	242.4±76.3^a^	132.0–373.0	227.0±112.9^a^	65.0–375.0	118.4±46.0^b^	43.0–244.0
ALKP (UL^-1^)	32.9±14.3	15.0–64.0	39.5±20.0	12.0–94.00 (21)	32.3±11.6	15.0–60.0
ALT (UL^-1^)	27.7±8.3	15.0–45.0 (12)	25.5±15.4	12.0–73.0 (17)	25.8±8.9	12.0–40.0 (16)
AST (UL^-1^)*	63.7±39.8^b^	28.0–146.0 (16)	97.6±92.5^b^	30.0–368.0 (22)	246.1±108.1ª	81.0–402.0
GGT (UL^-1^)	0.0±0.0	0.0–0.0	0.04±0.20	0.0–1.0	0.0±0.0	0.0–0.0
CK (UL^-1^)*	47.1±47.7^b^	0.0–127.0	104.4±195.7^b^	0.0–786.0 (22)	743.0±451.0^a^	101.0–1537.0 (13)
AMYL (UL^-1^)*	270.0±90.2^b^	89.0–380.0	274.1±69.0^b^	182.0–463.0	362.1±99.4ª	94.0–525.0
Calcium (mgdL^-1^)	3.6±2.4	0.1–7.8	4.6±3.3	0.0–10.7	5.6±2.1	1.9–9.8
Phosphorus (mgdL^-1^)	9.7±2.8	6.5–16.1	10.5±2.4	6.9–14.7 (18)	9.2±1.8	6.6–12.9 (19)

SCL = Straight carapace length, PCV = Packed cell volume, A/G = Albumin/Globulin, BUN = Blood urea nitrogen, AMYL = Amylase, ALKP = Alkaline phosphatase, ALT = Alanine aminotransferase, AST = Aspartate aminotransferase, GGT = Gamma-glutamyl transpeptidase, CK = Creatine phosphokinase. SD = Standard deviation

* = Significant difference

n^a^ = Number of samples analyzed if < n

differing letters indicate significant difference between groups.

## Discussion

Although olive ridley turtles are globally the most abundant sea turtle species and are subsequently at lower risk of extinction [33], knowledge regarding their population characteristics and health within foraging areas remains limited. Based on apparent age estimation methods, captured individuals represented subadult and adult turtles [21]. This resembles previously reported population structure for olive ridley turtles within foraging areas where all life stages are present. Although we did not capture juvenile turtles, their presence in the study area has been previously reported [34, 35].

Health indicators including BCI and physical examination have been used as a tool to determine the general health status of multiple free-living species [23, 36]. BCI is derived from the relationship between an animal´s length and weight resulting in an indirect estimate of sea turtle nutritional and health status [37]. Our study found similar BCI values to those reported in healthy populations of black turtles (*Chelonia mydas agassizii*) in the Gulf of California and along the western coast of Baja California’s foraging areas [38–41]. The Gulf of California has been previously identified as an important foraging habitat for multiple sea turtle species [15]. BCI values from individual turtles in this and previous studies indicate that these foraging areas present the necessary conditions to maintain healthy sea turtle populations. On the other hand, BCI was greater than that reported in Brazil [42] and in Venezuela [43] where the majority of turtles presented poor body condition including individuals with signs of physical illness and disease.

We found similar values in parameter as PCV, total protein, albumin, globulin and A/G ratio between our females turtles with healthy olive ridley turtles sampled during the breeding season in Costa Rica [44]. An increase in the concentration of plasma proteins has been observed when the turtles begin their breeding season [11]; however, this similarity may be due to the nutritional status of our turtles [45].

Parameters such as triglycerides, cholesterol, glucose, BUN, calcium and phosphorus are related to sea turtle nutritional status and diet [4, 10–12, 46]. In our foraging area, we found higher values of BUN and glucose than those reported in nesting areas [44, 47]. This difference is due of physiological anorexia caused by turtles mating and nesting in these studies [11, 48]. On the other hand, we found lower values of cholesterol, triglycerides and calcium in our adult females compared to nesting females of these same studies. Contrary to this, the values observed in males turtles were similar to those reported in the males sampled in Costa Rica [47]. This is due that during the reproduction, lipid and calcium concentrations increasing considerably in females due to vitellogenesis and folliculogenesis [49–51]. Similar results exist for other sea turtle species [12, 48, 52–54]. In addition, during nesting lipid and calcium mobilization increase to supply energy and muscle contraction respectively [49, 51]. Unfortunately, there are no studies that confirm the migratory routes and nesting sites of the population studied. Therefore, it is necessary to have multidisciplinary studies such as satellite telemetry and stable isotopes, for a better knowledge of the population.

Plasma enzymes are a good indicator of health since increased levels can signify organ-specific damage [55]. In this study, values of ALT and AST are higher than those reported on olive ridley sea turtle sampled during the breeding season [44, 47]. This may be due to the nutritional status of individuals in this study compared to the aforementioned studies, i.e., in the feeding grounds there is an increase in the activity of ALT and AST as they fulfill a metabolic function in the conversion of food (mainly protein) to energy. Anderson et al. (2011) observed an increase in the enzymatic activity of ALT, AST, ALKP and amylase during the postprandial stage, indicative of organs that are prepared for digestion, including the liver, pancreas and the gastrointestinal tract [29].

In relation to Amylase, CK and GGT, they are enzymes that have not been previously reported in olive ridley turtle. Amylase is an enzyme that participates in the digestion of food [29] and an excessive or insufficient amount could indicate a disorder of the pancreas [56]. Previous studies have shown that in sea turtles the values of this enzyme also tend to increase due to hormonal factors related to the nesting process [28, 53, 57]. CK is characterized by being a highly variable enzyme and is an important component of muscle metabolism [57], its increased in bloodstream is attributed to muscle damage [55]. Contrary to CK, GGT is found in low concentrations in sea turtles [55]; however, its increase in the bloodstream could indicate kidney or liver damage. Although the efficacy of blood biochemistry as a health indicator has been demonstrated, it is necessary to know all characteristics of the population to arrive at a more accurate conclusion [10].

Studies have suggested variations in blood parameters due to age and sex [9]. Santoro and Meneses (2007) evaluated the blood biochemistry of female and male olive ridley during breeding season and observed significant differences in parameters such as albumin, triglycerides, cholesterol, calcium, phosphorus, BUN and glucose [47], this is due to the vitellogenesis and folliculogenesis in females, and different foraging during this season. Contrary to this, we found no significant differences in blood parameters between female and male olive ridley turtles in the studied foraging area, which is consistent with results obtained in other sea turtle species [6, 9, 58]. Differences observed in PCV, total protein, albumin and A/G ratio between adults and subadults in our study could be associated with physiological changes due to growth and sexual maturity. For example, previous studies have reported that sea turtles present a positive correlation between PCV with age and size [48, 59–61]. On the other hand, when an organism enters reproductive age, plasma protein levels increase as a sign of sexual maturity [7, 50, 62].

Few studies have analysed the interannual differences of blood parameters [63]. In our study, the differences in parameters such as total protein, albumin and triglycerides could be due to the sexual maturity of turtles captured in each year. During 2013, the population was characterized by adults, while 2015 by subadults. On the other hand, we observed annual differences in CK, AST and glucose during the study period which could be a result of stress caused during capture. It has been shown that capture method [64] and handling during taking blood sample may influence parameters such as K, Ca, P, AST [63], triglycerides, albumin, GGT, LDH [65] and corticosterone [66]. On the other hand, these differences could also be due to environmental temperature [60]; however, the scope of the present study did not allow us to evaluate these factors, future work should consider these observations.

In addition to sex and age, body condition can be used as a reference to determine energy status. An animal presenting a good overall condition is assumed to have higher energy reserves than one in poor condition [37, 67]. Sea turtles have a high demand for energy during both migration and reproduction [49, 68]. Different studies have suggested a relationship between BCI and blood parameters as a reflection of sea turtle health status, with a positive correlation between BCI and total protein, triglycerides and glucose for proper nutrition and vice versa [42, 63]. We found no correlation between the BCI and analysed blood parameters as BCI did not change between turtles.

## Conclusions

To the best of our knowledge, this is the first study to establish blood biochemistry parameters for foraging olive ridley turtles. These values can be used as blood reference ranges for a healthy population of northern Sinaloa, Mexico olive ridley sea turtles. However, it is important to consider factors such as a turtle's age and sex when comparing blood parameters, since life stage is an influencing factor. Establish baseline health parameters for turtles in foraging and nesting habitats is a priority. This information will contribute to improved care for captive turtles and serve as a baseline for wild turtle health and monitoring.

## Supporting information

S1 Supporting Information Blood parameters from olive ridley sea turtles sampled for this study. (XLSX)

## References

[1] Swimmer JY. Biochemical responses to fibropapilloma and captivity in the green turtle. Journal of Wildlife Diseases. 2000;36(1):102–10. 10.7589/0090-3558-36.1.102 . 10682751

[2] Voigt GL, Swist SL. Hematology techniques and concepts for veterinary technicians2011.

[3] Greffe A, Friedrichs K, Harr K, Concordet D, Trumel C, Braun J. Reference values: a review. Veterinary Clinical Pathology. 2009;28(3):288–98. 10.1111/j.1939-165X.2009.00179.x 19737162

[4] Aguirre AA, Balazs GH. Blood biochemistry values of Green Turtle, *Chelonia mydas*, whith and whithout fibropapillomatosis. Comp Hematol Int. 2000;10:132–7.

[5] Hofer H, East ML. Stress and immunosupression as factors in the declive and extintion of wildlife population: concepts, evidence, and challenges In: Aguirre AA, Ostfeld RS, Daszak P, editors. New directions in conservation medicine: applied cases of ecological health. New York: Oxford University Press; 2012 p. 82–110.

[6] Ley-Quiñónez CP, Rossi-Lafferriere NA, Espinosa-Carreón TL, Hart CE, Peckham SH, Aguirre AA, et al Associations between trace elements and clinical health parameters in the North Pacific loggerhead sea turtle (*Caretta caretta*) from Baja California Sur, Mexico. Environ Sci Pollut Res Int. 2017 10.1007/s11356-017-8556-x . 28238183

[7] Delgado C, Valente A, Quaresma I, Costa M, Dellinger T. Blood biochemistry reference values for wild juvenile loggerhead sea turtles (*Caretta caretta*) from Madeira archipelago. Journal of Wildlife Diseases. 2011;47(3):523–9. 10.7589/0090-3558-47.3.523 . 21719817

[8] Flint M, Morton JM, Limpus CJ, Patterson-Kane JC, Mills PC. Reference intervals for plasma biochemical and hematologic measures in loggerhead sea turtles (*Caretta caretta*) from Moreton Bay, Australia. Journal of Wildlife Diseases. 2010;46(3):731–41. 10.7589/0090-3558-46.3.731 . 20688679

[9] Bolten AB, Bjorndal KA. Blood profiles for a wild population of green turtles (*Chelonia mydas*) in the southern Bahamas: size-specific and sex-specific relationships. Journal of Wildlife Diseases. 1992;28(3):407–13. 10.7589/0090-3558-28.3.407 . 1512872

[10] Whiting SD, Guinea ML, Limpus CJ, Fomiatti K. Blood chemistry reference values for two ecologically distinct populations of foraging green turtles, eastern Indian Ocean. Comp Clin Pathol. 2007;16:109–18.

[11] Deem SL, Norton TM, Mitchell M, Segars A, Alleman AR, Cray C, et al Comparison of blood values in foraging, nesting, and stranded loggerhead turtles (*Caretta caretta*) along the coast of Georgia, USA. Journal of Wildlife Diseases. 2009;45(1):41–56. 10.7589/0090-3558-45.1.41 . 19204334

[12] Ehsanpour M, Ahmadi MR, Bahri AH, Afkhami M, Reich KJ. Plasma biochemistry values in wild female hawksbill turtles (*Eretmochelys imbricata*), during nesting and foraging seasons in Qeshm Island, Persian Gulf. Comp Clin Pathol. 2015;24:561–6.

[13] Aguirre AA, Spraker TR, Chaves A, Du Toit L, Eure W, Balazs GH. Pathology of fibropapillomatosis in Olive Ridley turtles *Lepidochelys olivacea* nesting in Costa Rica. Journal of Aquatic Animal Health. 1999;11(3):283–9. 10.1577/1548-8667(1999)011<0283:Pofior>2.0.Co;2 PubMed PMID: WOS:000087184700010.

[14] Graciano-Obeso A. Caracterización ambiental de la zona de alimentación de tortugas marinas en el lirtoral del municipio de Guasave, Sinaloa [Master]. Guasave, Sinaloa: Instituto Politécnico Nacional; 2013.

[15] Seminoff JA. Sea Turtles of the Gulf of California In: Brusca RC, editor. The Gulf of California: Biodiversity and Conservation: The Arizona Board of Regents; 2010 p. 135–67.

[16] Zavala-Norzagaray AA, Ley-Quiñónez CP, Espinosa-Carreón TL, Canizalez-Román A, Hart CE, Aguirre AA. Trace elements in blood of sea turtles *Lepidochelys olivacea* in the Gulf of California, Mexico. Bull Environ Contam Toxicol. 2014;93(5):536–41. 10.1007/s00128-014-1320-8 24957795

[17] Zavala-Norzagaray AA. Medicina de Conservación (metales y microbiología) en Tortuga Marinas del Sistema Lagunar Navachiste y Zona de Influencia [PhD]. Culiacán, Sinaloa Universidad Autíonoma de Sinaloa; 2014.

[18] Valverde RA, Selcer KW, Lara LR, Sibaja-Cordero JA. Lack of xenoestrogen-induced vitellogenin in male olive ridley sea turtles (*Lepidochelys olivacea*) from the Pacific coast of Costa Rica. Rev Biol Trop 2008;56(5):49–57.

[19] Limpus CJ. A study of the loggerhead turtle, *Caretta caretta*, in eastern Australia [PhD]. Brisbane, Australia: University of Queensland; 1985.

[20] Bolten AB. Técnicas para la medición de tortugas marinas In: Eckert KL, Bjorndal KA, Abreu-Grobois FA, Donnelly M, editors. Técnicas de Investigación y Manejo para la Conservación de las Tortugas Marinas. 4 D.C: Grupo Especialista en Tortugas Marinas, UICN/CSE; 2000 p. 126–31.

[21] Márquez MR. Las Tortugas marinas y nuestro tiempo México D.F: Fondo de Cultura Económica; 1996. 197 p.

[22] Wilbbels T. Determinación del sexo de tortugas marinas en habitats de alimentación In: Eckert KL, Bjorndal KA, Abreu-Grobois FA, Donnelly M, editors. Técnicas de Investigación y Manejo para la Conservación de las Tortugas Marinas 4 D.C: Grupo Especialista en Tortugas Marinas, UICN/CSE 2000 p. 160–4.

[23] Thomson JA, Burkholder D, Heithaus MR, Dill LM. Validation of a rapid visual-assessment technique for categorizing the body condition of Green turtles (*Chelonia mydas*) in the Field. Copeia. 2009;2(2):251–5. 10.1643/Ce-07-227 PubMed PMID: WOS:000267151100007.

[24] Bjorndal KA. Prioridades para la investigación en hábitats de alimentación In: Eckert KAB K. L., Abreu-Grobois F. A. & Donnelly M., editor. Técnicas de Investigación y Manejo para la Conservación de las Tortugas Marinas. 4 D.C: Grupo Especialista en Tortugas Marinas, UICN/CSE; 2000 p. 13–5.

[25] Sikes IV JM, Klaphake E. Reptile hematology. Veterinary Clinics Exot Anim. 2008;11:481–500. 10.1016/j.cvex.2008.03.005 18675730

[26] Owens DW, Ruiz GJ. New methods of obtaining blood and cerebrospinal-fluid from marine turtles. Herpetologica. 1980;36(1):17–20. PubMed PMID: WOS:A1980JT27700003.

[27] Bolten AB, Jacobson ER, Bjorndal KA. Effects of anticoagulant and autoanalyzer on blood biochemical values of loggerhead sea turtles (*Caretta caretta*). American Journal of Veterinary Research. 1992;53(12):2224–7. . 1476302

[28] Camacho M, Oros J, Boada LD, Zaccaroni A, Silvi M, Formigaro C, et al Potential adverse effects of inorganic pollutants on clinical parameters of loggerhead sea turtles (*Caretta caretta*): results from a nesting colony from Cape Verde, West Africa. Mar Environ Res. 2013;92:15–22. 10.1016/j.marenvres.2013.08.002 . 23998796

[29] Anderson ET, Minter LJ, Clarke EO 3rd, Mroch RM, 3rd, Beasley JF, Harms CA. The Effects of Feeding on Hematological and Plasma Biochemical Profiles in Green (*Chelonia mydas*) and Kemp's Ridley (*Lepidochelys kempii*) Sea Turtles. Veterinary Medicine International. 2011:890829 10.4061/2011/890829 ; PubMed Central PMCID: PMCPMC3135279. 21776356 PMC3135279

[30] IDEXX. IDEXX VetTest Chemistry analyzer operator's manual. Maine, USA: 2014.

[31] Stamper MA, Harms C, Epperly SP, Braun-McNeill J, Avens L, Stoskopf MK. Relationship between barnacle epibiotic load and hematologic parameters in loggerhead sea turtles (*Caretta caretta*), a comparison between migratory and residential animals in Pamlico Sound, North Carolina. Journal of Zoo and Wildlife Medicine. 2005;36(4):635–41. 10.1638/04-074.1 PubMed PMID: WOS:000236593400009. 17312720

[32] Norton TM, Wyneken J. Body Condition Scoring the Sea Turtle: Lafeber Vet; 2015 [cited 2017 20 april]. Available from: http://lafeber.com/vet/body-condition-scoring-the-sea-turtle/.

[33] Abreu-Grobois A, Plotkin P. *Lepidochelys olivacea*-Red List Of Threatened Species 2014 [cited 2017 15 may]. Available from: www.Iucnredlist.Org.

[34] Zavala A, Briseño R, Ramos M, Aguirre A. First record of juvenile olive ridley (*Lepidochelys olivacea*) in Northern Sinaloa Gulf of California, México. 27 Annual Symposium on Sea Turtle Biology and Conservation; Myrntle Beach, South Carolina. 2007.

[35] Aguilar-González ME. Evaluación del impacto antropogénico sobre las poblaciones de tortuga marina en la zona costera del sistema lagunar San Ignacio-Macapule-Navachiste, Sinaloa, México [Master]. Guasave, Sinaloa: Instituto Politécnico Nacional; 2009.

[36] Jakob EM, Marshall SD, Uetz GW. Estimating fitness: A comparison of body condition indices. Oikos. 1996;77(1):61–7. 10.2307/3545585 PubMed PMID: WOS:A1996VQ98600007.

[37] Stevenson RD, Woods WA. Condition indices for conservation: new uses for evolving tools. Integr Comp Biol. 2006;46(6):1169–90. 10.1093/icb/icl052 21672816

[38] Seminoff JA, Jones TT, Resendiz A, Nichols WJ, Chaloupka MY. Monitoring green turtles (*Chelonia mydas*) at a coastal foraging area in Baja California, Mexico: multiple indices describe population status. J Mar Biol Ass UK 2003;83:1355–62.

[39] Labrada-Martagon V, Mendez-Rodriguez LC, Gardner SC, Cruz-Escalona VH, Zenteno-Savin T. Health Indices of the Green Turtle (*Chelonia mydas*) Along the Pacific Coast of Baja California Sur, Mexico. II. Body Condition Index. Chelonian Conservation and Biology. 2010;9(2):173–83. 10.2744/Ccb-0807.1 PubMed PMID: WOS:000285966500004.

[40] López-Castro MC, Koch V, Mariscal-Loza A, Nichols WJ. Long-term monitoring of black turtles *Chelonia mydas* at coastal foraging areas off the Baja California Peninsula. Endangered Species Research. 2010;11:35–45. 10.3354/esr00264

[41] Najera-Hillman E, Bass JB, Buckham S. Distribution patterns of the barnacle, *Chelonibia testudinaria*, on juvenile green turtles (*Chelonia mydas*) in Bahia Magdalena, Mexico. Revista Mexicana De Biodiversidad. 2012;83(4):1171–9. 10.7550/rmb.27444 PubMed PMID: WOS:000313932200022.

[42] de Deus Santos MR, Silva Martins A, Baptistotte C, Work TM. Health condition of juvenile *Chelonia mydas* related to fibropapillomatosis in southeast Brazil. Diseases of Aquatic Organisms. 2015;115(3):193–201. 10.3354/dao02883 . 26290504

[43] Barrios-Garrido H, Espinoza-Rodriguez N, Shimaa T, Wildermann NE. Body condition index in rescued Green turtles (*Chelonia mydas*) in the Gulf of Venezuela: a seven year assesment. 35th Annual Symposium on Sea Turtle Biology and Conservation; Turkey: MACART press; 2015.

[44] Brenes-Chaves L, Berrocal A, Meneses A, Jiménez Sánchez C, Orrego Vásquez CM. Study on the etiology of fibropapillomatosis of olive ridley sea turtles (*Lepidochelys olivacea*) nesting in the National Wildlife Refuge at Ostional, Guanacaste, Costa Rica. Rev Mar Cost. 2013;5:16. Epub 2013-12-15.

[45] Muñoz-Peréz JP, Lewbart GA, Hirschfeld M, Alarcon-Ruales D, Denkinger J, Castaneda JG, et al Blood gases, biochemistry and haematology of Galapagos hawksbill turtles (*Eretmochelys imbricata*). Conserv Physiol. 2017;5(1):cox028 10.1093/conphys/cox028 ; PubMed Central PMCID: PMCPMC5424066. 28496982 PMC5424066

[46] Anderson ET, Harms CA, Stringer EM, Cluse WM. Evaluation of hematology and serum biochemistry of cold-stunned green sea turtles (*Chelonia mydas*) in North Carolina, USA. Journal of Zoo and Wildlife Medicine. 2011;42(2):247–55. 10.1638/2010-0217.1 22946402

[47] Santoro M, Meneses A. Haematology and plasma chemistry of breeding olive ridley sea turtles (*Lepidochelys olivacea*). Veterinary Record 2007;161(24):818–9. . 18083982

[48] Casal AB, Camacho M, Lopez-Jurado LF, Juste C, Oros J. Comparative study of hematologic and plasma biochemical variables in Eastern Atlantic juvenile and adult nesting loggerhead sea turtles (*Caretta caretta*). Veterinary Clinical Pathology / American Society for Veterinary Clinical Pathology. 2009;38(2):213–8. 10.1111/j.1939-165X.2008.00106.x . 19192261

[49] Hamann M, Limpus CJ, Whittier JM. Patterns of lipid storage and mobilisation in the female green sea turtle (*Chelonia mydas*). J Comp Physiol B. 2002;172(6):485–93. 10.1007/s00360-002-0271-2 . 12192510

[50] Hamann M, Limpus CJ, Owens DW. Reproductive cycles of males and females In: Lutz P. L., Musick J. A., Wyneken J, editors. The biology of sea turtles. 2 Boca Raton: CRC Press; 2003 p. 135–98.

[51] Price ER. The physiology of lipid storage and use in reptiles. Biological Reviews. 2016. 10.1111/brv.12288 27348513

[52] Deem SL, Dierenfeld ES, Sounguet GP, Alleman AR, Cray C, Poppenga RH, et al Blood values in free-ranging nesting leatherback sea turtles (*Dermochelys coriacea*) on the coast of the Republic of Gabon. Journal of Zoo and Wildlife Medicine: official publication of the American Association of Zoo Veterinarians. 2006;37(4):464–71. 10.1638/05-102.1 . 17315430

[53] Goldberg DW, Wanderlinde J, Freire IMA, da Silva LCP, Almosny NRP. Serum biochemistry profile determination for wild loggerhead sea turtles nesting in Campos dos Goytacazes, Rio de Janeiro, Brazil. Cienc Rural. 2011;41(1):143–8. PubMed PMID: WOS:000287990900023.

[54] Prieto-Torres DA, Hernandez JL, Henriquez AR, Alvarado MC, Davila MJ. Blood Biochemistry of the Breeding Population of Green Turtles (*Chelonia mydas*) in the Aves Island Wildlife Refuge, Venezuela. South American Journal of Herpetology. 2013;8(3):147–54. 10.2994/Sajh-D-13-00010.1 PubMed PMID: WOS:000329195000001.

[55] Anderson ET, Socha VL, Gardner J, Byrd L, Manire CA. Tissue enzyme activities in the loggerhead sea turtle (*Caretta caretta*). Journal of Zoo and Wildlife Medicine. 2013;44(1):62–9. 10.1638/1042-7260-44.1.62 . 23505704

[56] Núñez L, Bouda J. Patología Clínica Veterinaria. 2 ed: Facultad de Medicina Veterinaria y Zootecnia UNAM, México, D.F.; 2007. 334 p.

[57] Perrault JR, Miller DL, Eads E, Johnson C, Merrill A, Thompson LJ, et al Maternal Health Status Correlates with Nest Success of Leatherback Sea Turtles (*Dermochelys coriacea*) from Florida. PLOS ONE. 2012;7(2):e31841 10.1371/journal.pone.0031841 22359635 PMC3281022

[58] Flint M, Morton JM, Limpus CJ, Patterson-Kane JC, Murray PJ, Mills PC. Development and application of biochemical and haematological reference intervals to identify unhealthy green sea turtles (*Chelonia mydas*). Vet J. 2010;185(3):299–304. 10.1016/j.tvjl.2009.06.011 PubMed PMID: WOS:000281476100013. 19709912

[59] Kakizoe Y, Sakaoka K, Kakizoe F, Yoshii M, Nakamura H, Kanou Y, et al Successive changes of hematologic characteristics and plasma chemistry values of juvenile loggerhead turtles (*Caretta caretta*). Journal of Zoo and Wildlife Medicine: official publication of the American Association of Zoo Veterinarians. 2007;38(1):77–84. 10.1638/05-096.1 . 17469279

[60] Fong CL, Chen HC, Cheng IJ. Blood profiles from wild populations of green sea turtles in Taiwan. Journal of Veterinary Medicine and Animal Health. 2010;2(2):8–10.

[61] Rousselet E, Stacy NI, LaVictoire K, Higgins BM, Tocidlowski ME, Flanagan JP, et al Hematology and plasma biochemistry analytes in five age groups of immature, captive-reared loggerhead sea turtles (*Caretta caretta*). Journal of Zoo and Wildlife Medicine: official publication of the American Association of Zoo Veterinarians. 2013;44(4):859–74. 10.1638/2012-0162R1.1 . 24450044

[62] Fazio E, Liotta A, Medica P, Giacoppo E, Ferlazzo A. Effects of different health status on blood haematochemical values of loggerhead sea turtles (*Caretta caretta*). Comp Clin Pathol 2012;21:105–9.

[63] Labrada-Martagón V, Méndez-Rodríguez LC, Gardner SC, López-Castro M, Zenteno-Savín T. Health Indices of the Green Turtle (*Chelonia mydas*) Along the Pacific Coast of Baja California Sur, Mexico. I. Blood Biochemistry Values. Chelonian Conservation and Biology. 2010;9(2):162–72. 10.2744/ccb-0806.1

[64] Snoddy JE, Landon M, Blanvillain G, Southwood A. Blood biochemistry of sea turtles captured in gillnets in the Lower Cape Fear River, North Carolina, USA. Journal of Wildlife Management. 2009;73(8):1394–401. 10.2193/2008-472 PubMed PMID: WOS:000271437400018.

[65] Aguirre AA, Balazs GH, Spraker TR, Gross TS. Adrenal and hematological responses to stress in juvenile green turtles (*Chelonia mydas*) with and without fibropapillomas. Physiological Zoology. 1995;68(5):831–54.

[66] Gregory LF, Schmid JR. Stress responses and sexing of wild Kemp's ridley sea turtles (*Lepidochelys kempii*) in the northeastern Gulf of Mexico. Gen Comp Endocrinol. 2001;124(1):66–74. 10.1006/gcen.2001.7683 . 11703072

[67] Schulte-Hostedde AI, Zinner B, Millar JS, Hickling GJ. Restitution of Mass-Size Residuals: Validating Body Condition Indices. Ecology. 2005;86(1):155–63.

[68] Jessop TS, Hamann M, Limpus CJ. Body condition and physiological changes in male green turtles during breeding. Marine Ecology Progress Series. 2004;276:281–8.

